# Item

**Published:** 1901-07

**Authors:** 


### ITEM.

THE Congress on Tuberculosis will be held at London, July 23-26, 1901. The British Medical Association will meet at Cheltenham, July 29-August 2, 1901. A medical excursion is planning to attend these meetings under the management of H. Gaze & Sons, 113 Broadway, New York. The cost for a twenty-nine day trip is $238.00. The itinery is as follows: July 10th, leave New York by Red Star Line, new twin screw express steamer Zeeland; July 18th, due at Cherbourg and proceed to Paris. (Oceanic Hotel); July 19th, Paris, to July 22d; leaving Paris evening of 22d for London; July 23d to July 28th, London. St. Ermin's Hotel; July 29th, at Cheltenham, to August 1st; sail August 1st from Liverpool by Ss. New England; August 8th, due at Boston.

